# Cyclo­(-l-prolyl-l-valinyl-) from *Burkholderia thailandensis* MSMB43

**DOI:** 10.1107/S1600536812043000

**Published:** 2012-10-20

**Authors:** Xiang-Yang Liu, Cheng Wang, Yi-Qiang Cheng

**Affiliations:** a Department of Biological Sciences, Department of Chemistry and Biochemistry, University of Wisconsin–Milwaukee, PO Box 413, Milwaukee, WI 53201, USA

## Abstract

The title compound [systematic name: (3*S*,8a*S*)-3-isopropyl­hexa­hydro­pyrrolo­[1,2-*a*]pyrazine-1,4-dione], C_10_H_16_N_2_O_2_,, is a newly isolated cyclic dipeptide from *Burkholderia thailandensis* MSMB43. There are two independent mol­ecules in the asymmetric unit. Two C atoms and their attached H atoms in the five-membered ring of one of the mol­ecules are disordered over two sets of sites in a 0.715 (5):0.285 (5) ratio. The two independent mol­ecules have the same configuration and the absolute configurations of the chiral centers were determined based on the observation of anomalous dispersion. In the crystal, two types of N—H⋯O hydrogen bonds link pairs of independent mol­ecules.

## Related literature

For general background to secondary metabolites from *B. thailandensis*, see: Knappe *et al.* (2008 ▶); Nguyen *et al.* (2008 ▶); Seyedsayamdost *et al.* (2010 ▶); Ishida *et al.* (2010 ▶); Klausmeyer *et al.* (2011 ▶); Biggins *et al.* (2011 ▶); Wang *et al.* (2011 ▶, 2012 ▶); Ishida *et al.* (2012 ▶). For isolation of the title compound from other microorganisms, see: Chen (1960 ▶); Schmitz *et al.* (1983 ▶); Jayatilake *et al.* (1996 ▶); Ginz & Engelhardt (2000 ▶); Qi *et al.* (2009 ▶); Wang *et al.* (2010 ▶); Park *et al.* (2006 ▶). For the biological activity of the title compound, see: Holden *et al.* (1999 ▶); Fdhila *et al.* (2003 ▶). For large-scale genome sequencing, see: Mukhopadhyay *et al.* (2010 ▶); Yu *et al.* (2006 ▶); Zhuo *et al.* (2012 ▶). For our work on obtaining natural products from *B. thailandensis* MSMB43, see: Liu *et al.* (2012 ▶).
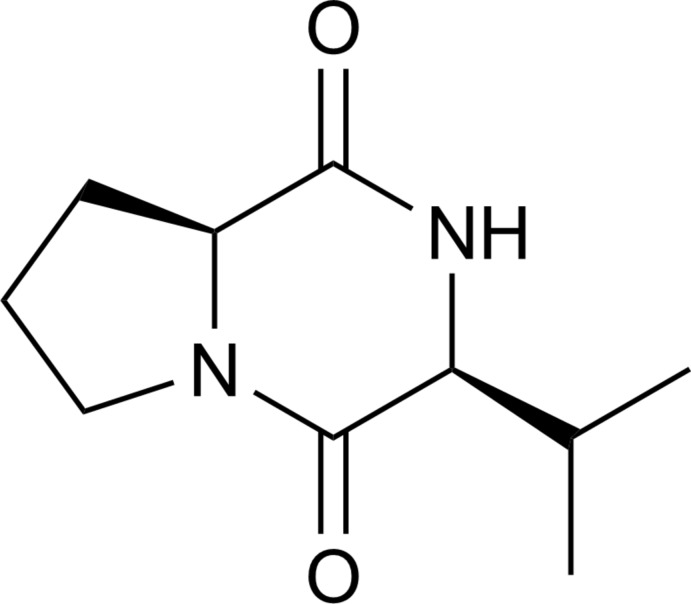


## Experimental

### 

#### Crystal data

C_10_H_16_N_2_O_2_
*M* *_r_* = 196.25
Orthorhombic, 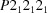

*a* = 5.6227 (1) Å
*b* = 10.2571 (2) Å
*c* = 34.2115 (6) Å
*V* = 1973.07 (6) Å^3^
*Z* = 8
Cu *K*α radiation
μ = 0.76 mm^−1^
*T* = 100 K
0.22 × 0.14 × 0.10 mm

#### Data collection

Bruker APEXII area-detector diffractometer
Absorption correction: multi-scan (*SADABS*; Bruker, 2007 ▶) *T*_min_ = 0.851, *T*_max_ = 0.928
28285 measured reflections
3668 independent reflections
3354 reflections with *I* > 2σ(*I*)
*R*_int_ = 0.045

#### Refinement

*R*[*F* ^2^ > 2σ(*F* ^2^)] = 0.031
*wR*(*F* ^2^) = 0.075
*S* = 1.02
3668 reflections
271 parameters
3 restraints
H atoms treated by a mixture of independent and constrained refinement
Δρ_max_ = 0.30 e Å^−3^
Δρ_min_ = −0.23 e Å^−3^
Absolute structure: Flack (1983 ▶), 1481 Friedel pairs
Flack parameter: 0.05 (17)

### 

Data collection: *APEX2* (Bruker, 2007 ▶); cell refinement: *SAINT* (Bruker, 2007 ▶); data reduction: *SAINT*; program(s) used to solve structure: *SHELXTL* (Sheldrick, 2008 ▶); program(s) used to refine structure: *SHELXTL*; molecular graphics: *SHELXTL* and *OLEX2* (Dolomanov *et al.*, 2009 ▶); software used to prepare material for publication: *SHELXTL* and *OLEX2*.

## Supplementary Material

Crystal structure: contains datablock(s) global, I. DOI: 10.1107/S1600536812043000/ff2084sup1.cif

Supplementary material file. DOI: 10.1107/S1600536812043000/ff2084Isup2.cdx

Structure factors: contains datablock(s) I. DOI: 10.1107/S1600536812043000/ff2084Isup3.hkl

Supplementary material file. DOI: 10.1107/S1600536812043000/ff2084Isup4.cml

Additional supplementary materials:  crystallographic information; 3D view; checkCIF report

## Figures and Tables

**Table 1:** Hydrogen-bond geometry (Å, °)

*D*—H⋯*A*	*D*—H	H⋯*A*	*D*⋯*A*	*D*—H⋯*A*
N1—H1⋯O1*A* ^i^	0.872 (19)	2.016 (19)	2.8734 (17)	167.7 (17)
N1*A*—H1*A*⋯O1^ii^	0.916 (19)	2.06 (2)	2.9710 (17)	172.3 (17)

Symmetry codes: (i) 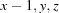; (ii) 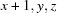.

**Fig. 1.**
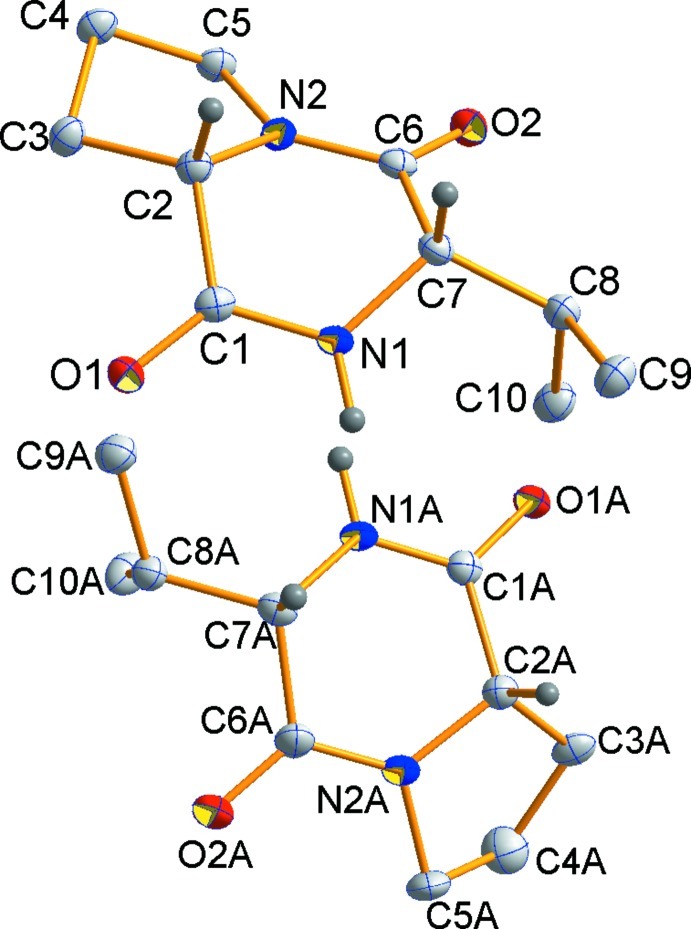
A molecular structures of cyclo(-L-prolyl-L-valinyl-) in asymmetric unit with displacement ellipsoids shown at the 50% probability level. All hydrogen atoms attached to non-chiral carbon atoms and minor components of disordered atoms were omitted for clarity.

**Fig. 2.**
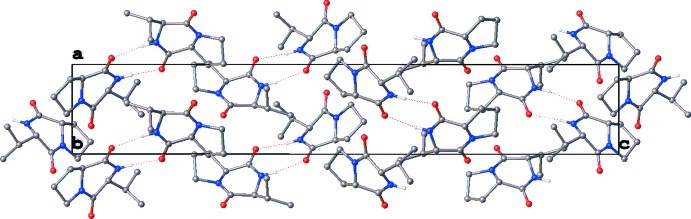
A packing diagram of cyclo(-L-prolyl-L-valinyl-), viewed along the b axis. For clarity, all H atoms attached to carbon atoms are omitted. The dashed lines represent hydrogen bonds.

## supplementary crystallographic information

### Comment

Many interesting compounds, including thailandamides A—B (Ishida *et al.*, 2012, Nguyen *et al.*, 2008, Ishida *et al.*, 2010), capistruin (Knappe *et al.*, 2008), bactobolins A—D (Seyedsayamdost *et al.*, 2010), burkholdacs A—B (Biggins *et al.*, 2011), spiruchostatin C (Klausmeyer *et al.*, 2011) and thailandepsins A—F (Wang *et al.*, 2011, Wang *et al.*, 2012), were discovered from *Burkholderia thailandensis* E264 in recent years. In conjunction with large-scale genome sequencing (Mukhopadhyay *et al.*, 2010, Zhuo *et al.*, 2012, Yu *et al.*, 2006), the *Burkholderia* species have drawn much attention due to their capabilities to produce novel compounds with antibacterial, antitumor and antiviral activities. As a result of our expanded natural product discovery from *Burkholderia* species, we have recently confirmed that *B. thailandensis* MSMB43 can produce high titers of FK228 in M8 medium (Liu *et al.*, 2012). Here we report the crystal structure of a known dipeptide isolated from the culture broth of *B. thailandensis* MSMB43 grown in M11 medium.

The title compound is a cyclic dipeptide of *L*-proline and *L*-valine. The structural skeleton is fused by a five-membered pyrrolidine ring and a six-membered piperazine ring. The pyrrolidine ring adopts an envelope configuration and the piperazine ring has a boat configuration. These two rings are located on nearly the same plane and the dihedral angles of these two least-squares planes are 18.2 (1)° for the non-disordered molecule, and 30.6 (1)° for the major component of the disordered molecule. There are two independent molecules in the asymmetric unit of the crystal. Atoms C3A and C4A of one of the molecules are disordered over two positions with a major component contribution of 71.5 (5)%. The two molecules have the same configuration and the absolute configurations of C2, C2A, C7 and C7A are *S* based on the results of anomalous dispersion. There are two intermolecular hydrogen bonds present between two independent molecules in the different asymmetric unit and connect them to form a pair of molecules (Table 1, Fig. 1 and Fig. 2).

### Experimental

*Isolation of the title compound Bacterial strain and culture conditions B. thailandensis* strain MSMB43 was obtained from the US Centers for Disease Control (CDC) and was routinely activated on LB agar containing 50 mg ml^-1^ of apramycin (Am^50^) at 37°C for 1 to 2 days as a master plate. A single colony was then transferred into a 1-*L* flask containing 300 ml of LB medium and Am^50^, and the culture were growing at 37°C for 24 h as seed culture. For fed-batch fermentation 250 ml of seed culture was transferred into a 20-*L* fermentor (BioFlo IV, New Brunswick Scientific Co., USA) containing 12 *L* of M11 medium (10.0 g/*L* dextrose, 2.0 g/*L* pancreatic digest of casein, 1.0 g/*L* yeast extract, 1.0 g/*L* beef extract; pH 7.0). Fermentation was proceeded at 37°C, 300 rpm for 72 h, during which the pH was automatically adjusted by the fermentor with 1 N HCl or 1 N NaOH. Three liters of 10X M11 was fed to the fermentor from 24 h to 48 h at a flow rate of 0.125 ml/min.

*Recovery of the crude extract*

Bacterial cells and debris were removed by centrifugation of broth at 6,000 g for 15 min. Supernatant was applied to a 2-*L* column (*Φ* 8.0 *x* 40 cm) packed with a 50/50 mixture of Diaion HP-20 resin (Sigma-Aldrich, USA) and Amberlite XAD16 resin (Sigma-Aldrich) to allow absorption to occur. The resins were subsequently dried and extracted repeatedly with ethyl acetate. Organic extractions were pooled and dried in a rotary evaporator to yield a crude extract.

*Isolation and purification of the title compound*

Crude extract was mixed with 50 g silica gel (230–400 mesh, Whatman Purasil, USA). The mixture of silica gel was dried overnight and then applied to a 120 - g silica gel column, which has been equilibrated with hexane. The column was eluted sequentially with 1*L* of hexane (fraction 1), 1*L* of hexane:ethyl acetate (3:1, *v*/*v*) (fraction 2), 1*L* of hexane:ethyl acetate (1:1, *v*/*v*) (fraction 3), 1*L* of ethyl acetate (fraction 4), 1*L* of ethyl acetate:acetone (1:1, *v*/*v*) (fraction 5) and 2*L* of acetone (fraction 6). Fraction 5 was applied on a flash chromatography equipped with a silica gel Universal Column (*Φ* 23 ×123 mm, 16 g, Yamazen Corporation,) mounted atop an injection column (*Φ* 20 × 65 mm, 14 g, Yamazen Corporation). The column was eluted by mixtures of chloroform and acetone with increasing polarity according to the following scheme: 1%, 5%, 10%, 15%, 20%, 25%, 30%, 35%, 65%, 100% of acetone. Fraction eluted by 10% acetone was applied on a preparative HPLC system equipped with an Agilent Prep-C18 column (*Φ* 21.2 × 250 mm, 10 µm). The mobile phase consists of acetonitrile and water. The column was first eluted by 10% acetonitrile for 90 min, then by a gradient from 10% to 15% acetronitrile from 90 min to 100 min, then by 15% acetonitrile for 30 min, and finally by 100% acetonitrile for 20 min. The flow rate was 8 ml/min. The UV spectrum was monitored at 210 nm. The title compound was eluted at 30.0 min and other compounds were eluted at later times.

*Crystallization*

The purified title compound was dissolved in ethyl acetate and the crystals were obtained after a slow evaporation of the solvent at room temperature for 5 days.

### Refinement

All hydrogen atoms attached to the carbon atoms were placed in geometrically idealized positions (C—H = 0.98, 0.99 and 1.00 Å on the primary, secondary and tertiary aliphatic C atoms respectively). The H atoms were refined as riding, with isotropic displacement coefficients of *U*_iso_(H) = 1.5*U*_eq_(C) for methyl groups or 1.2*U*_eq_(C) otherwise. The hydrogen atoms attached to N were located in the difference map and refined independently with restraints and constraints. The H atoms on the N were constrained to have the distances of 0.88 Å and the *U*_iso_ value were assigned as equal to 1.2 times the *U*_eq_ of the attached atoms.

### Crystal data

**Table:**

C_10_H_16_N_2_O_2_	*F*(000) = 848
*M**_r_* = 196.25	*D*_x_ = 1.321 Mg m^−^^3^
Orthorhombic, *P*2_1_2_1_2_1_	Cu *K*α radiation, λ = 1.54178 Å
Hall symbol: P 2ac 2ab	Cell parameters from 999 reflections
*a* = 5.6227 (1) Å	θ = 2.6–69.5°
*b* = 10.2571 (2) Å	µ = 0.76 mm^−^^1^
*c* = 34.2115 (6) Å	*T* = 100 K
*V* = 1973.07 (6) Å^3^	Needle, colourless
*Z* = 8	0.22 × 0.14 × 0.10 mm

### Data collection

**Table:**

Bruker APEXII area-detector diffractometer	3668 independent reflections
Radiation source: fine-focus sealed tube	3354 reflections with *I* > 2σ(*I*)
Graphite monochromator	*R*_int_ = 0.045
0.50° ω and 0.5 ° φ scans	θ_max_ = 69.5°, θ_min_ = 2.6°
Absorption correction: multi-scan (*SADABS*; Bruker, 2007)	*h* = −6→6
*T*_min_ = 0.851, *T*_max_ = 0.928	*k* = −12→12
28285 measured reflections	*l* = −40→41

### Refinement

**Table:**

Refinement on *F*^2^	Secondary atom site location: difference Fourier map
Least-squares matrix: full	Hydrogen site location: inferred from neighbouring sites
*R*[*F*^2^ > 2σ(*F*^2^)] = 0.031	H atoms treated by a mixture of independent and constrained refinement
*wR*(*F*^2^) = 0.075	*w* = 1/[σ^2^(*F*_o_^2^) + (0.0411*P*)^2^ + 0.438*P*] where *P* = (*F*_o_^2^ + 2*F*_c_^2^)/3
*S* = 1.02	(Δ/σ)_max_ = 0.001
3668 reflections	Δρ_max_ = 0.30 e Å^−^^3^
271 parameters	Δρ_min_ = −0.23 e Å^−^^3^
3 restraints	Absolute structure: Flack (1983), 1481 Friedel pairs
Primary atom site location: structure-invariant direct methods	Flack parameter: 0.05 (17)

### Special details

**Table:**

Geometry. All e.s.d.'s (except the e.s.d. in the dihedral angle between two l.s. planes) are estimated using the full covariance matrix. The cell e.s.d.'s are taken into account individually in the estimation of e.s.d.'s in distances, angles and torsion angles; correlations between e.s.d.'s in cell parameters are only used when they are defined by crystal symmetry. An approximate (isotropic) treatment of cell e.s.d.'s is used for estimating e.s.d.'s involving l.s. planes.
Refinement. Refinement of *F*^2^ against ALL reflections. The weighted *R*-factor *wR* and goodness of fit *S* are based on *F*^2^, conventional *R*-factors *R* are based on *F*, with *F* set to zero for negative *F*^2^. The threshold expression of *F*^2^ > σ(*F*^2^) is used only for calculating *R*-factors(gt) *etc*. and is not relevant to the choice of reflections for refinement. *R*-factors based on *F*^2^ are statistically about twice as large as those based on *F*, and *R*- factors based on ALL data will be even larger.

### Fractional atomic coordinates and isotropic or equivalent isotropic displacement parameters (Å^2^)

**Table:**

	*x*	*y*	*z*	*U*_iso_*/*U*_eq_	Occ. (<1)
O1	−0.0686 (2)	0.57786 (10)	0.42809 (3)	0.0185 (2)	
O2	0.6845 (2)	0.29334 (10)	0.46538 (3)	0.0189 (2)	
N1	0.1607 (2)	0.39828 (12)	0.41718 (4)	0.0158 (3)	
H1	0.120 (3)	0.3984 (17)	0.3926 (6)	0.019*	
N2	0.4056 (2)	0.44278 (12)	0.48284 (4)	0.0151 (3)	
C1	0.0698 (3)	0.49203 (15)	0.43957 (4)	0.0154 (3)	
C2	0.1570 (3)	0.48683 (15)	0.48164 (4)	0.0152 (3)	
H2	0.0559	0.4246	0.4968	0.018*	
C3	0.1676 (3)	0.61678 (15)	0.50308 (5)	0.0177 (3)	
H3A	0.0138	0.6373	0.5157	0.021*	
H3B	0.2109	0.6886	0.4851	0.021*	
C4	0.3622 (3)	0.59338 (16)	0.53340 (4)	0.0179 (3)	
H4A	0.4259	0.6767	0.5436	0.021*	
H4B	0.3025	0.5404	0.5555	0.021*	
C5	0.5497 (3)	0.51970 (15)	0.51009 (4)	0.0167 (3)	
H5A	0.6562	0.5804	0.4960	0.020*	
H5B	0.6463	0.4628	0.5272	0.020*	
C6	0.4837 (3)	0.34091 (14)	0.46205 (4)	0.0155 (3)	
C7	0.2933 (3)	0.28844 (14)	0.43410 (4)	0.0155 (3)	
H7	0.1787	0.2360	0.4500	0.019*	
C8	0.3954 (3)	0.19864 (15)	0.40270 (4)	0.0175 (3)	
H8	0.4958	0.1321	0.4163	0.021*	
C9	0.1969 (3)	0.12534 (16)	0.38147 (5)	0.0215 (3)	
H9A	0.1029	0.1870	0.3660	0.032*	
H9B	0.0940	0.0825	0.4007	0.032*	
H9C	0.2668	0.0596	0.3641	0.032*	
C10	0.5556 (3)	0.27055 (17)	0.37385 (5)	0.0218 (3)	
H10A	0.4603	0.3323	0.3586	0.033*	
H10B	0.6305	0.2075	0.3562	0.033*	
H10C	0.6790	0.3180	0.3882	0.033*	
O1A	1.0544 (2)	0.43773 (11)	0.33589 (3)	0.0214 (2)	
O2A	0.4323 (2)	0.77776 (12)	0.27683 (3)	0.0320 (3)	
N1A	0.7596 (2)	0.58471 (13)	0.34599 (4)	0.0186 (3)	
H1A	0.799 (3)	0.5861 (18)	0.3720 (6)	0.022*	
N2A	0.6766 (3)	0.60603 (14)	0.26780 (4)	0.0221 (3)	
C1A	0.8856 (3)	0.50385 (15)	0.32357 (4)	0.0172 (3)	
C2A	0.8103 (3)	0.49229 (16)	0.28135 (4)	0.0190 (3)	
H2A	0.7014	0.4155	0.2795	0.023*	0.715 (5)
H2B	0.7274	0.4081	0.2756	0.023*	0.285 (5)
C3A	1.0062 (5)	0.4726 (3)	0.25090 (7)	0.0201 (8)	0.715 (5)
H3C	0.9797	0.3906	0.2363	0.024*	0.715 (5)
H3D	1.1637	0.4680	0.2638	0.024*	0.715 (5)
C4A	0.9952 (6)	0.5904 (3)	0.22307 (7)	0.0283 (7)	0.715 (5)
H4C	1.0389	0.5656	0.1960	0.034*	0.715 (5)
H4D	1.1001	0.6619	0.2320	0.034*	0.715 (5)
C3B	1.0460 (10)	0.5041 (11)	0.25504 (12)	0.0201 (8)	0.285 (5)
H3E	1.1567	0.5726	0.2642	0.024*	0.285 (5)
H3F	1.1306	0.4201	0.2518	0.024*	0.285 (5)
C4B	0.9032 (14)	0.5445 (9)	0.21869 (9)	0.0283 (7)	0.285 (5)
H4E	1.0131	0.5846	0.1996	0.034*	0.285 (5)
H4F	0.8355	0.4652	0.2065	0.034*	0.285 (5)
C5A	0.7235 (3)	0.62958 (18)	0.22612 (5)	0.0281 (4)	
H5C	0.6982	0.7222	0.2191	0.034*	0.715 (5)
H5D	0.6228	0.5738	0.2093	0.034*	0.715 (5)
H5E	0.7743	0.7210	0.2220	0.034*	0.285 (5)
H5F	0.5790	0.6131	0.2104	0.034*	0.285 (5)
C6A	0.5464 (3)	0.68536 (16)	0.28998 (5)	0.0218 (4)	
C7A	0.5431 (3)	0.65377 (15)	0.33383 (4)	0.0187 (3)	
H7A	0.4041	0.5954	0.3390	0.022*	
C8A	0.5085 (3)	0.77939 (15)	0.35758 (4)	0.0189 (3)	
H8A	0.3758	0.8289	0.3450	0.023*	
C9A	0.4322 (3)	0.75127 (17)	0.39963 (5)	0.0234 (4)	
H9D	0.5601	0.7050	0.4133	0.035*	
H9E	0.2885	0.6972	0.3995	0.035*	
H9F	0.3990	0.8336	0.4131	0.035*	
C10A	0.7283 (3)	0.86638 (17)	0.35596 (5)	0.0262 (4)	
H10D	0.6927	0.9504	0.3683	0.039*	
H10E	0.7740	0.8805	0.3286	0.039*	
H10F	0.8595	0.8241	0.3699	0.039*	

### Atomic displacement parameters (Å^2^)

**Table:**

	*U*^11^	*U*^22^	*U*^33^	*U*^12^	*U*^13^	*U*^23^
O1	0.0189 (5)	0.0192 (5)	0.0175 (5)	0.0049 (5)	−0.0028 (5)	−0.0005 (4)
O2	0.0154 (5)	0.0211 (5)	0.0203 (5)	0.0032 (5)	−0.0027 (5)	−0.0004 (5)
N1	0.0175 (7)	0.0179 (6)	0.0119 (6)	0.0029 (6)	−0.0035 (5)	0.0006 (5)
N2	0.0145 (6)	0.0170 (6)	0.0138 (6)	0.0008 (5)	−0.0021 (5)	−0.0011 (5)
C1	0.0140 (7)	0.0174 (7)	0.0148 (7)	−0.0030 (7)	−0.0006 (6)	0.0004 (6)
C2	0.0138 (7)	0.0172 (7)	0.0147 (7)	0.0002 (6)	0.0006 (6)	0.0001 (6)
C3	0.0151 (7)	0.0201 (7)	0.0178 (7)	0.0007 (6)	−0.0001 (6)	−0.0028 (6)
C4	0.0160 (8)	0.0216 (7)	0.0161 (7)	−0.0018 (7)	−0.0014 (6)	−0.0019 (6)
C5	0.0162 (7)	0.0184 (7)	0.0155 (7)	−0.0020 (7)	−0.0034 (6)	0.0001 (6)
C6	0.0173 (8)	0.0152 (7)	0.0139 (7)	−0.0016 (6)	−0.0007 (6)	0.0036 (6)
C7	0.0169 (8)	0.0145 (7)	0.0151 (7)	0.0005 (6)	−0.0001 (6)	0.0025 (6)
C8	0.0189 (8)	0.0167 (7)	0.0169 (7)	0.0023 (6)	−0.0022 (6)	−0.0004 (6)
C9	0.0243 (8)	0.0211 (8)	0.0190 (8)	−0.0011 (7)	0.0001 (7)	−0.0048 (7)
C10	0.0191 (8)	0.0266 (8)	0.0198 (8)	0.0015 (7)	0.0021 (7)	−0.0020 (7)
O1A	0.0228 (6)	0.0242 (6)	0.0173 (5)	0.0066 (5)	−0.0020 (5)	0.0014 (5)
O2A	0.0418 (7)	0.0346 (7)	0.0197 (6)	0.0179 (6)	−0.0074 (6)	0.0000 (5)
N1A	0.0206 (7)	0.0212 (7)	0.0139 (6)	0.0020 (6)	−0.0046 (5)	−0.0001 (6)
N2A	0.0271 (7)	0.0247 (7)	0.0146 (6)	0.0064 (6)	−0.0050 (6)	−0.0003 (5)
C1A	0.0181 (8)	0.0158 (7)	0.0177 (7)	−0.0024 (6)	−0.0013 (6)	0.0010 (6)
C2A	0.0209 (8)	0.0191 (7)	0.0169 (8)	0.0005 (7)	−0.0020 (7)	0.0001 (7)
C3A	0.0151 (12)	0.0287 (18)	0.0164 (9)	−0.0045 (11)	−0.0045 (9)	−0.0028 (9)
C4A	0.0290 (18)	0.0287 (16)	0.0271 (11)	0.0011 (12)	0.0122 (11)	0.0109 (11)
C3B	0.0151 (12)	0.0287 (18)	0.0164 (9)	−0.0045 (11)	−0.0045 (9)	−0.0028 (9)
C4B	0.0290 (18)	0.0287 (16)	0.0271 (11)	0.0011 (12)	0.0122 (11)	0.0109 (11)
C5A	0.0412 (11)	0.0307 (9)	0.0124 (8)	0.0064 (8)	−0.0042 (7)	0.0000 (7)
C6A	0.0223 (9)	0.0243 (8)	0.0187 (8)	0.0034 (8)	−0.0070 (7)	−0.0028 (7)
C7A	0.0174 (8)	0.0204 (8)	0.0182 (7)	0.0004 (6)	−0.0025 (7)	0.0026 (6)
C8A	0.0191 (8)	0.0210 (8)	0.0166 (7)	0.0030 (7)	−0.0003 (6)	0.0006 (6)
C9A	0.0219 (8)	0.0287 (9)	0.0197 (8)	0.0035 (7)	0.0021 (7)	0.0002 (7)
C10A	0.0331 (10)	0.0219 (8)	0.0236 (8)	−0.0068 (8)	0.0048 (8)	−0.0012 (7)

### Geometric parameters (Å, º)

**Table:**

O1—C1	1.2390 (19)	N2A—C6A	1.332 (2)
O2—C6	1.2354 (19)	N2A—C2A	1.463 (2)
N1—C1	1.331 (2)	N2A—C5A	1.470 (2)
N1—C7	1.4698 (19)	C1A—C2A	1.510 (2)
N1—H1	0.872 (19)	C2A—C3A	1.530 (3)
N2—C6	1.3381 (19)	C2A—C3B	1.607 (4)
N2—C5	1.4656 (19)	C2A—H2A	1.0000
N2—C2	1.469 (2)	C2A—H2B	1.0000
C1—C2	1.521 (2)	C3A—C4A	1.539 (3)
C2—C3	1.523 (2)	C3A—H3C	0.9900
C2—H2	1.0000	C3A—H3D	0.9900
C3—C4	1.527 (2)	C4A—C5A	1.583 (4)
C3—H3A	0.9900	C4A—H4C	0.9900
C3—H3B	0.9900	C4A—H4D	0.9900
C4—C5	1.523 (2)	C3B—C4B	1.537 (3)
C4—H4A	0.9900	C3B—H3E	0.9900
C4—H4B	0.9900	C3B—H3F	0.9900
C5—H5A	0.9900	C4B—C5A	1.359 (7)
C5—H5B	0.9900	C4B—H4E	0.9900
C6—C7	1.533 (2)	C4B—H4F	0.9900
C7—C8	1.527 (2)	C5A—H5C	0.9900
C7—H7	1.0000	C5A—H5D	0.9900
C8—C10	1.526 (2)	C5A—H5E	0.9900
C8—C9	1.529 (2)	C5A—H5F	0.9900
C8—H8	1.0000	C6A—C7A	1.535 (2)
C9—H9A	0.9800	C7A—C8A	1.536 (2)
C9—H9B	0.9800	C7A—H7A	1.0000
C9—H9C	0.9800	C8A—C10A	1.525 (2)
C10—H10A	0.9800	C8A—C9A	1.528 (2)
C10—H10B	0.9800	C8A—H8A	1.0000
C10—H10C	0.9800	C9A—H9D	0.9800
O1A—C1A	1.2402 (19)	C9A—H9E	0.9800
O2A—C6A	1.230 (2)	C9A—H9F	0.9800
N1A—C1A	1.333 (2)	C10A—H10D	0.9800
N1A—C7A	1.469 (2)	C10A—H10E	0.9800
N1A—H1A	0.916 (19)	C10A—H10F	0.9800
			
C1—N1—C7	121.45 (13)	N2A—C2A—C3B	100.7 (3)
C1—N1—H1	116.9 (12)	C1A—C2A—C3B	107.4 (3)
C7—N1—H1	121.0 (12)	C3A—C2A—C3B	15.0 (4)
C6—N2—C5	125.23 (13)	N2A—C2A—H2A	107.1
C6—N2—C2	122.52 (13)	C1A—C2A—H2A	107.1
C5—N2—C2	112.22 (12)	C3A—C2A—H2A	107.1
O1—C1—N1	124.88 (14)	N2A—C2A—H2B	112.8
O1—C1—C2	121.83 (13)	C1A—C2A—H2B	112.7
N1—C1—C2	113.28 (13)	C3B—C2A—H2B	109.8
N2—C2—C1	110.11 (12)	C2A—C3A—C4A	106.77 (18)
N2—C2—C3	102.62 (12)	C2A—C3A—H3C	110.4
C1—C2—C3	115.95 (13)	C4A—C3A—H3C	110.4
N2—C2—H2	109.3	C2A—C3A—H3D	110.4
C1—C2—H2	109.3	C4A—C3A—H3D	110.4
C3—C2—H2	109.3	H3C—C3A—H3D	108.6
C2—C3—C4	102.58 (13)	C3A—C4A—C5A	101.4 (2)
C2—C3—H3A	111.3	C3A—C4A—H4C	111.5
C4—C3—H3A	111.3	C5A—C4A—H4C	111.5
C2—C3—H3B	111.3	C3A—C4A—H4D	111.5
C4—C3—H3B	111.3	C5A—C4A—H4D	111.5
H3A—C3—H3B	109.2	H4C—C4A—H4D	109.3
C5—C4—C3	102.61 (12)	C4B—C3B—C2A	92.4 (3)
C5—C4—H4A	111.2	C4B—C3B—H3E	113.2
C3—C4—H4A	111.2	C2A—C3B—H3E	113.2
C5—C4—H4B	111.2	C4B—C3B—H3F	113.2
C3—C4—H4B	111.2	C2A—C3B—H3F	113.2
H4A—C4—H4B	109.2	H3E—C3B—H3F	110.6
N2—C5—C4	102.57 (12)	C5A—C4B—C3B	114.2 (4)
N2—C5—H5A	111.3	C5A—C4B—H4E	108.7
C4—C5—H5A	111.3	C3B—C4B—H4E	108.7
N2—C5—H5B	111.3	C5A—C4B—H4F	108.7
C4—C5—H5B	111.3	C3B—C4B—H4F	108.7
H5A—C5—H5B	109.2	H4E—C4B—H4F	107.6
O2—C6—N2	124.02 (14)	C4B—C5A—N2A	102.1 (2)
O2—C6—C7	123.85 (14)	N2A—C5A—C4A	101.27 (15)
N2—C6—C7	112.11 (13)	N2A—C5A—H5C	111.5
N1—C7—C8	112.07 (12)	C4A—C5A—H5C	111.5
N1—C7—C6	109.31 (12)	N2A—C5A—H5D	111.5
C8—C7—C6	112.84 (13)	C4A—C5A—H5D	111.5
N1—C7—H7	107.5	H5C—C5A—H5D	109.3
C8—C7—H7	107.5	C4B—C5A—H5E	111.5
C6—C7—H7	107.5	N2A—C5A—H5E	110.3
C10—C8—C7	112.67 (13)	C4B—C5A—H5F	113.5
C10—C8—C9	111.19 (13)	N2A—C5A—H5F	110.7
C7—C8—C9	110.87 (13)	H5E—C5A—H5F	108.7
C10—C8—H8	107.3	O2A—C6A—N2A	123.30 (15)
C7—C8—H8	107.3	O2A—C6A—C7A	120.94 (14)
C9—C8—H8	107.3	N2A—C6A—C7A	115.76 (14)
C8—C9—H9A	109.5	N1A—C7A—C6A	111.64 (13)
C8—C9—H9B	109.5	N1A—C7A—C8A	111.08 (13)
H9A—C9—H9B	109.5	C6A—C7A—C8A	109.98 (13)
C8—C9—H9C	109.5	N1A—C7A—H7A	108.0
H9A—C9—H9C	109.5	C6A—C7A—H7A	108.0
H9B—C9—H9C	109.5	C8A—C7A—H7A	108.0
C8—C10—H10A	109.5	C10A—C8A—C9A	111.84 (14)
C8—C10—H10B	109.5	C10A—C8A—C7A	111.64 (13)
H10A—C10—H10B	109.5	C9A—C8A—C7A	112.04 (13)
C8—C10—H10C	109.5	C10A—C8A—H8A	107.0
H10A—C10—H10C	109.5	C9A—C8A—H8A	107.0
H10B—C10—H10C	109.5	C7A—C8A—H8A	107.0
C1A—N1A—C7A	125.26 (13)	C8A—C9A—H9D	109.5
C1A—N1A—H1A	116.0 (12)	C8A—C9A—H9E	109.5
C7A—N1A—H1A	118.0 (12)	H9D—C9A—H9E	109.5
C6A—N2A—C2A	126.07 (13)	C8A—C9A—H9F	109.5
C6A—N2A—C5A	123.44 (14)	H9D—C9A—H9F	109.5
C2A—N2A—C5A	110.25 (13)	H9E—C9A—H9F	109.5
O1A—C1A—N1A	123.44 (14)	C8A—C10A—H10D	109.5
O1A—C1A—C2A	119.79 (14)	C8A—C10A—H10E	109.5
N1A—C1A—C2A	116.76 (13)	H10D—C10A—H10E	109.5
N2A—C2A—C1A	112.62 (13)	C8A—C10A—H10F	109.5
N2A—C2A—C3A	105.04 (15)	H10D—C10A—H10F	109.5
C1A—C2A—C3A	117.37 (17)	H10E—C10A—H10F	109.5
			
C7—N1—C1—O1	−170.29 (14)	C5A—N2A—C2A—C3A	18.7 (2)
C7—N1—C1—C2	11.1 (2)	C6A—N2A—C2A—C3B	−141.0 (4)
C6—N2—C2—C1	−45.57 (18)	C5A—N2A—C2A—C3B	33.5 (4)
C5—N2—C2—C1	136.54 (13)	O1A—C1A—C2A—N2A	−158.55 (15)
C6—N2—C2—C3	−169.60 (13)	N1A—C1A—C2A—N2A	22.8 (2)
C5—N2—C2—C3	12.51 (15)	O1A—C1A—C2A—C3A	−36.4 (2)
O1—C1—C2—N2	−143.76 (14)	N1A—C1A—C2A—C3A	145.00 (17)
N1—C1—C2—N2	34.95 (18)	O1A—C1A—C2A—C3B	−48.6 (4)
O1—C1—C2—C3	−27.8 (2)	N1A—C1A—C2A—C3B	132.8 (4)
N1—C1—C2—C3	150.87 (14)	N2A—C2A—C3A—C4A	7.3 (3)
N2—C2—C3—C4	−33.24 (14)	C1A—C2A—C3A—C4A	−118.7 (3)
C1—C2—C3—C4	−153.30 (13)	C2A—C3A—C4A—C5A	−27.8 (3)
C2—C3—C4—C5	42.04 (15)	N2A—C2A—C3B—C4B	−40.4 (7)
C6—N2—C5—C4	−164.35 (13)	C1A—C2A—C3B—C4B	−158.4 (5)
C2—N2—C5—C4	13.47 (16)	C2A—C3B—C4B—C5A	41.1 (9)
C3—C4—C5—N2	−33.83 (15)	C3B—C4B—C5A—N2A	−23.0 (8)
C5—N2—C6—O2	5.3 (2)	C6A—N2A—C5A—C4B	166.1 (4)
C2—N2—C6—O2	−172.35 (14)	C2A—N2A—C5A—C4B	−8.6 (5)
C5—N2—C6—C7	−175.76 (13)	C6A—N2A—C5A—C4A	138.76 (19)
C2—N2—C6—C7	6.64 (19)	C2A—N2A—C5A—C4A	−35.9 (2)
C1—N1—C7—C8	−175.93 (14)	C3A—C4A—C5A—N2A	37.7 (3)
C1—N1—C7—C6	−50.06 (19)	C2A—N2A—C6A—O2A	−177.98 (16)
O2—C6—C7—N1	−142.09 (14)	C5A—N2A—C6A—O2A	8.2 (3)
N2—C6—C7—N1	38.92 (17)	C2A—N2A—C6A—C7A	1.2 (2)
O2—C6—C7—C8	−16.7 (2)	C5A—N2A—C6A—C7A	−172.63 (15)
N2—C6—C7—C8	164.34 (13)	C1A—N1A—C7A—C6A	−31.1 (2)
N1—C7—C8—C10	56.61 (17)	C1A—N1A—C7A—C8A	−154.21 (15)
C6—C7—C8—C10	−67.31 (17)	O2A—C6A—C7A—N1A	−154.28 (15)
N1—C7—C8—C9	−68.72 (16)	N2A—C6A—C7A—N1A	26.5 (2)
C6—C7—C8—C9	167.36 (13)	O2A—C6A—C7A—C8A	−30.5 (2)
C7A—N1A—C1A—O1A	−172.92 (14)	N2A—C6A—C7A—C8A	150.31 (15)
C7A—N1A—C1A—C2A	5.6 (2)	N1A—C7A—C8A—C10A	53.83 (17)
C6A—N2A—C2A—C1A	−26.9 (2)	C6A—C7A—C8A—C10A	−70.28 (17)
C5A—N2A—C2A—C1A	147.63 (14)	N1A—C7A—C8A—C9A	−72.51 (17)
C6A—N2A—C2A—C3A	−155.8 (2)	C6A—C7A—C8A—C9A	163.39 (14)

### Hydrogen-bond geometry (Å, º)

**Table:**

*D*—H···*A*	*D*—H	H···*A*	*D*···*A*	*D*—H···*A*
N1—H1···O1*A*^i^	0.872 (19)	2.016 (19)	2.8734 (17)	167.7 (17)
N1*A*—H1*A*···O1^ii^	0.916 (19)	2.06 (2)	2.9710 (17)	172.3 (17)

Symmetry codes: (i) *x*−1, *y*, *z*; (ii) *x*+1, *y*, *z*.
